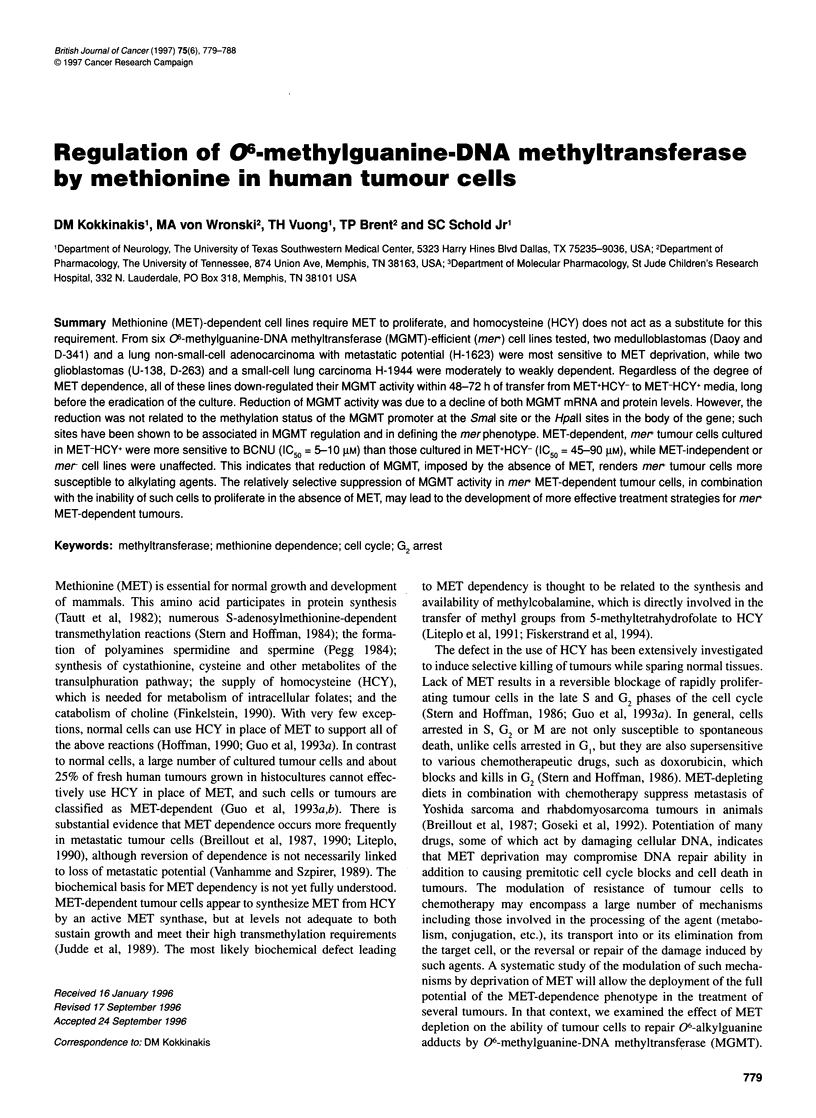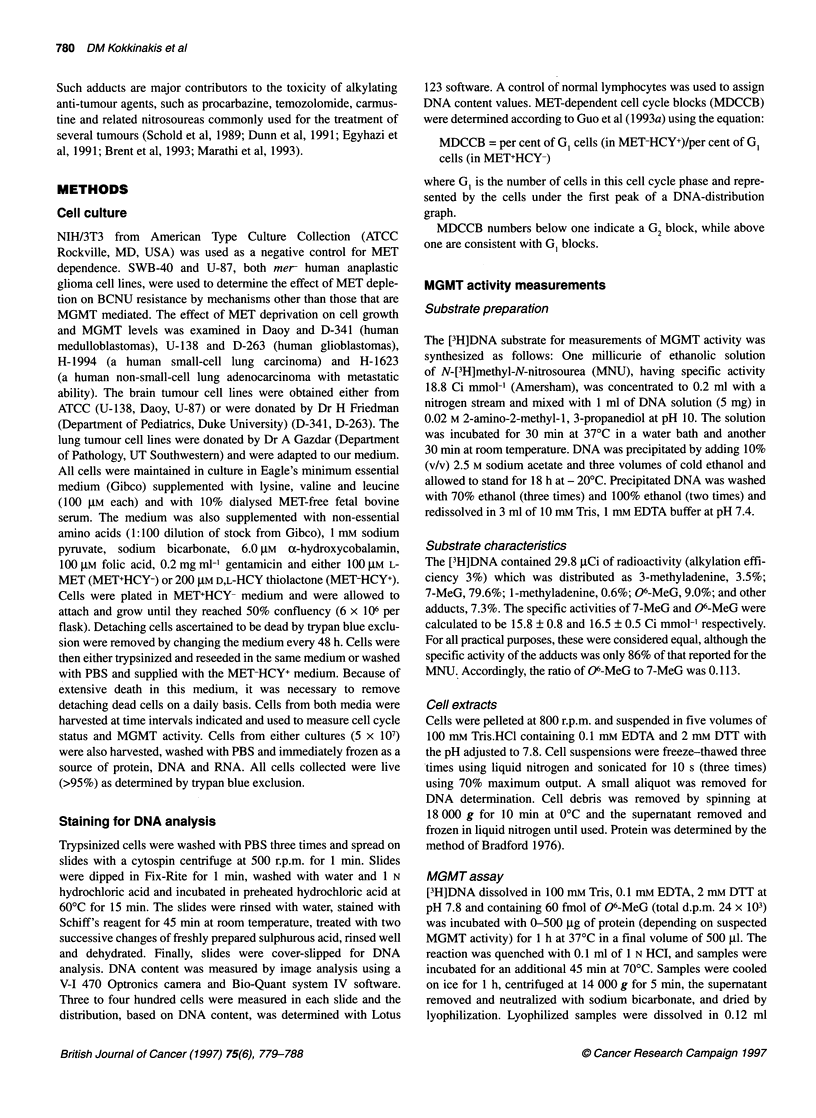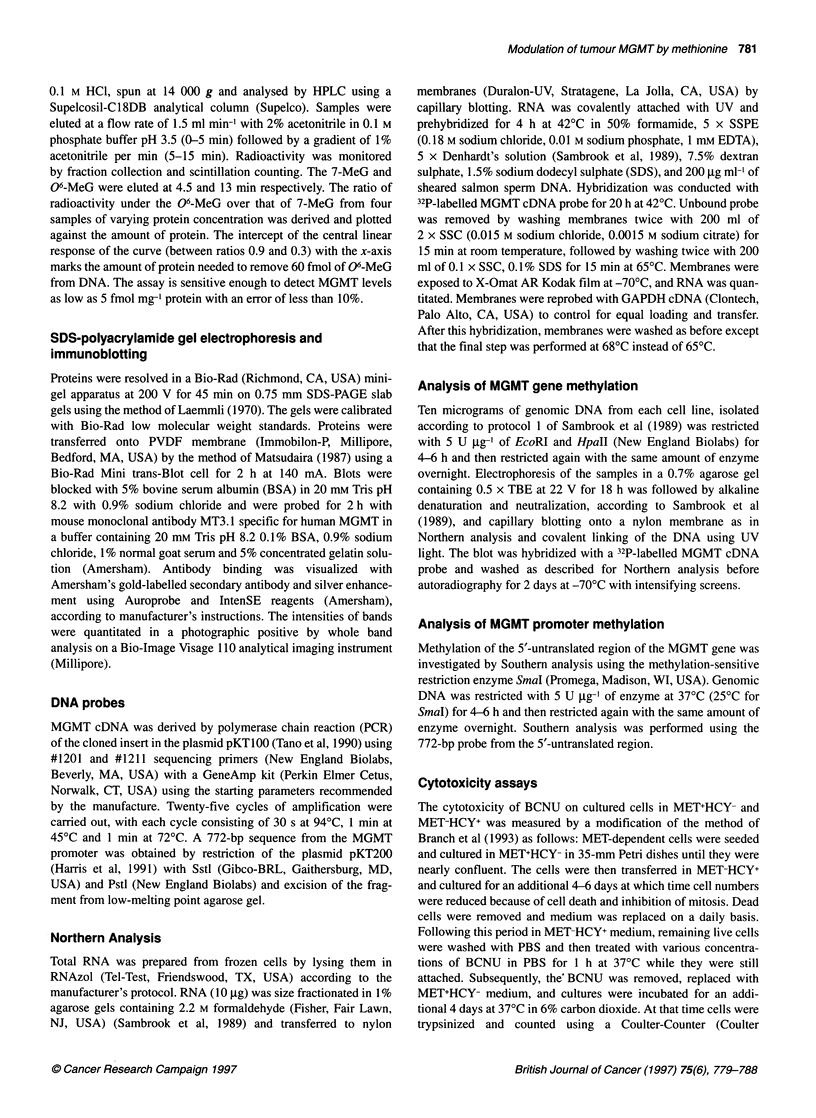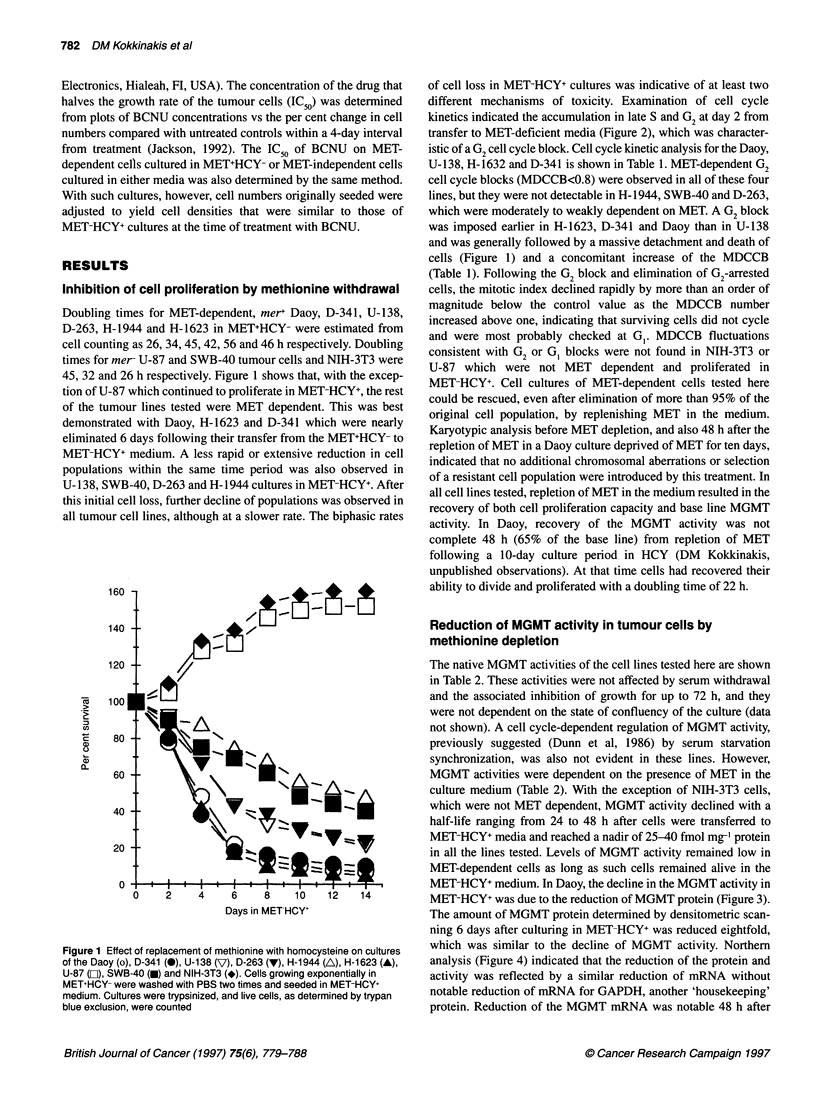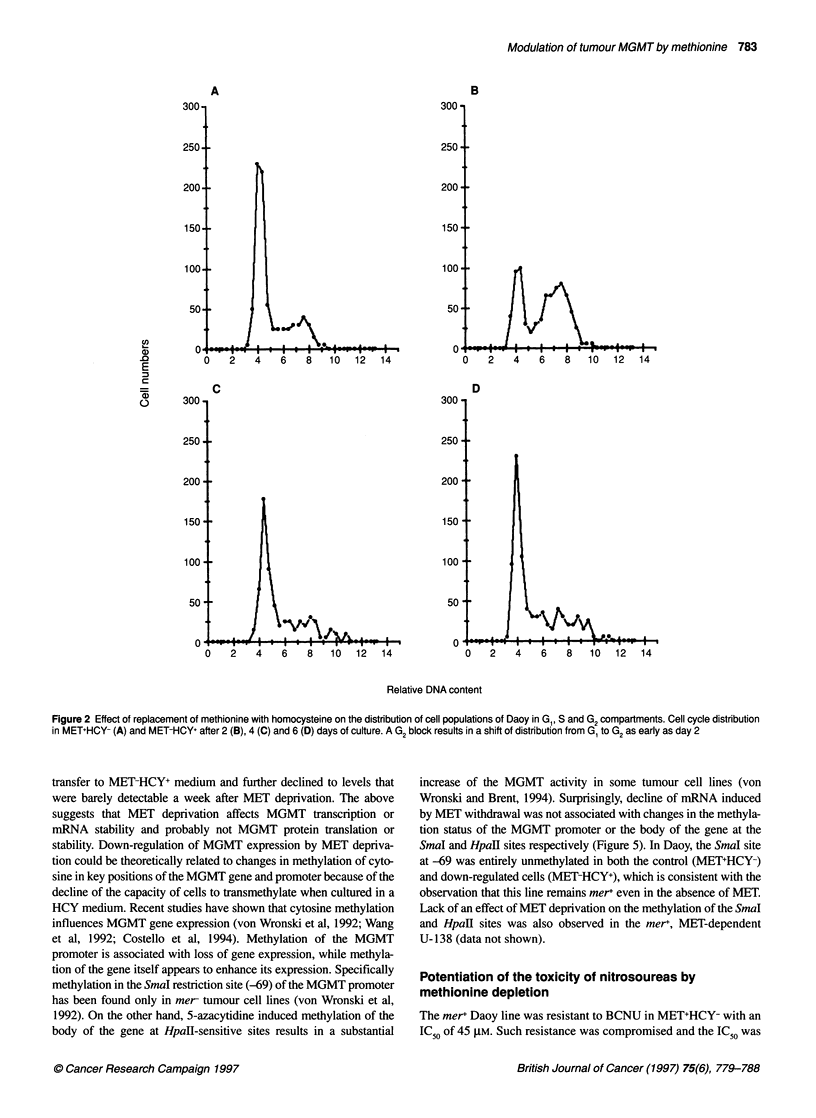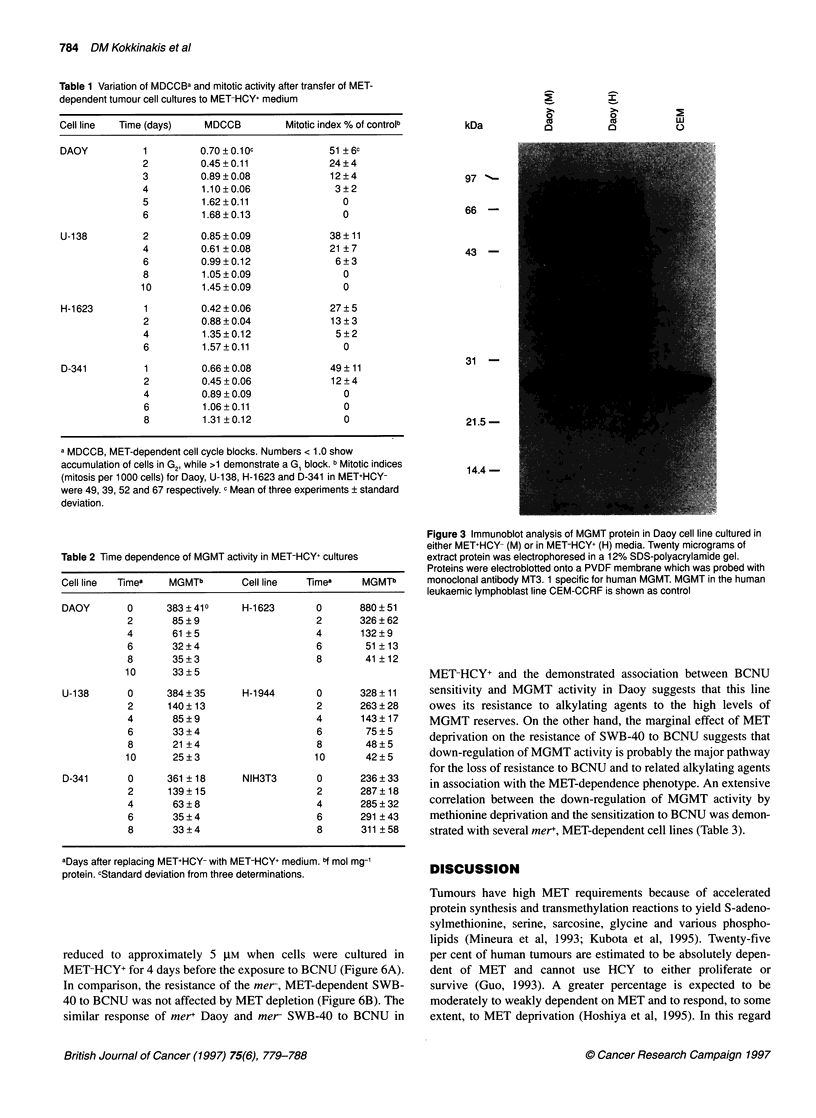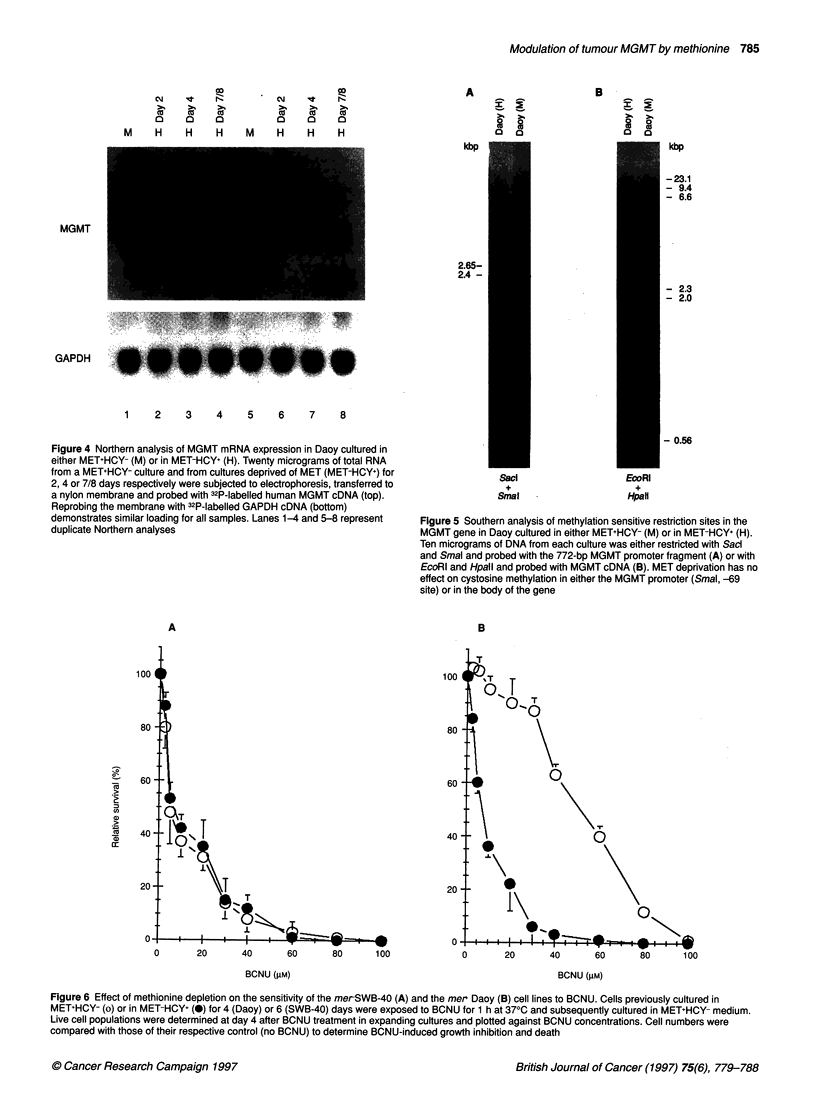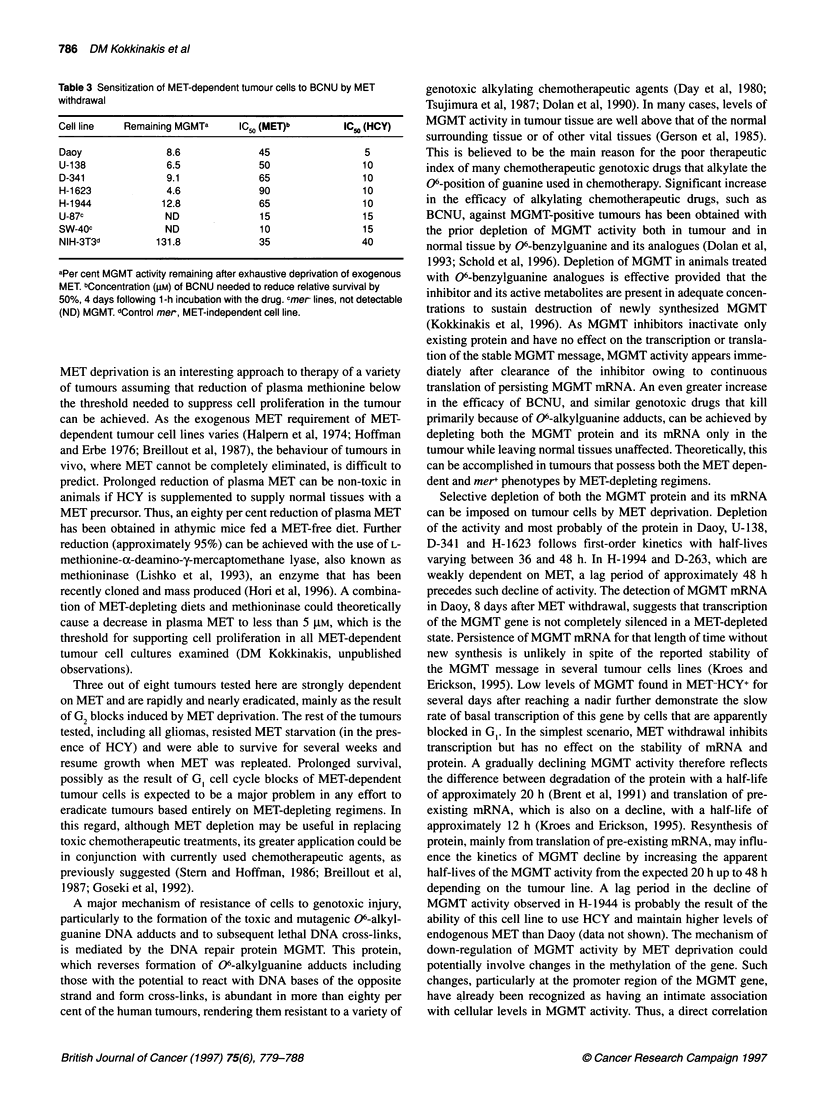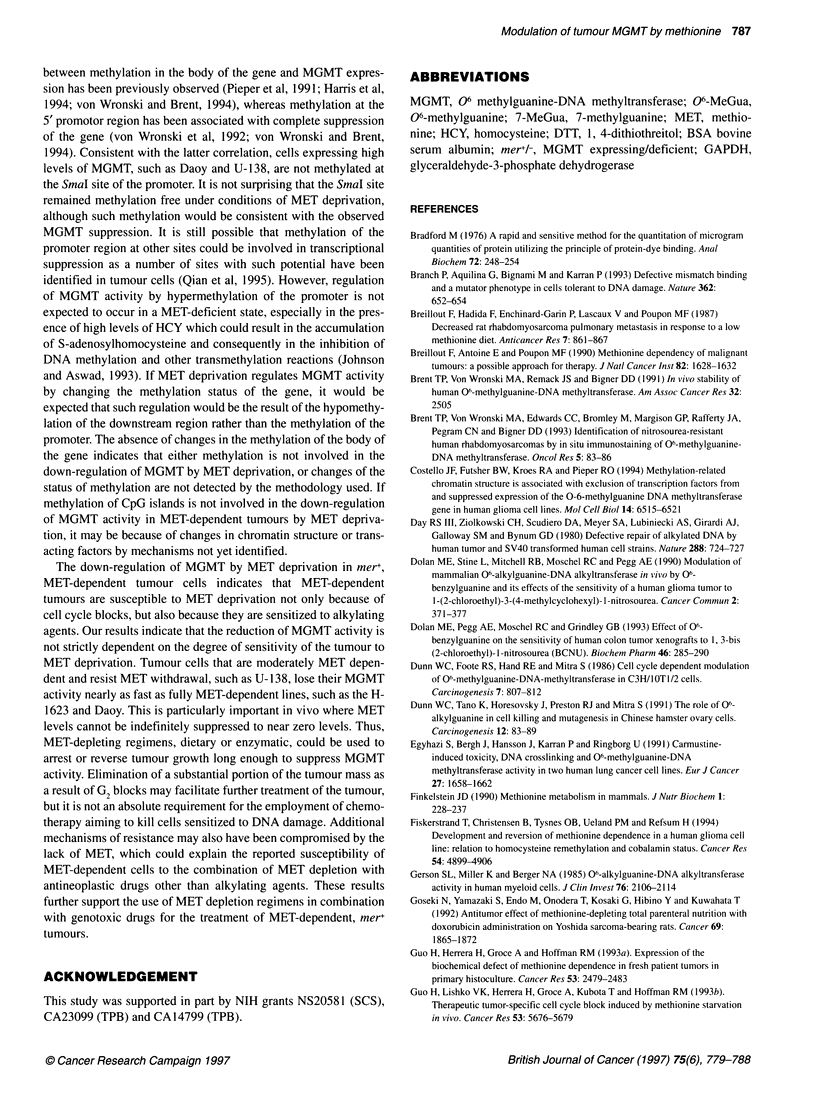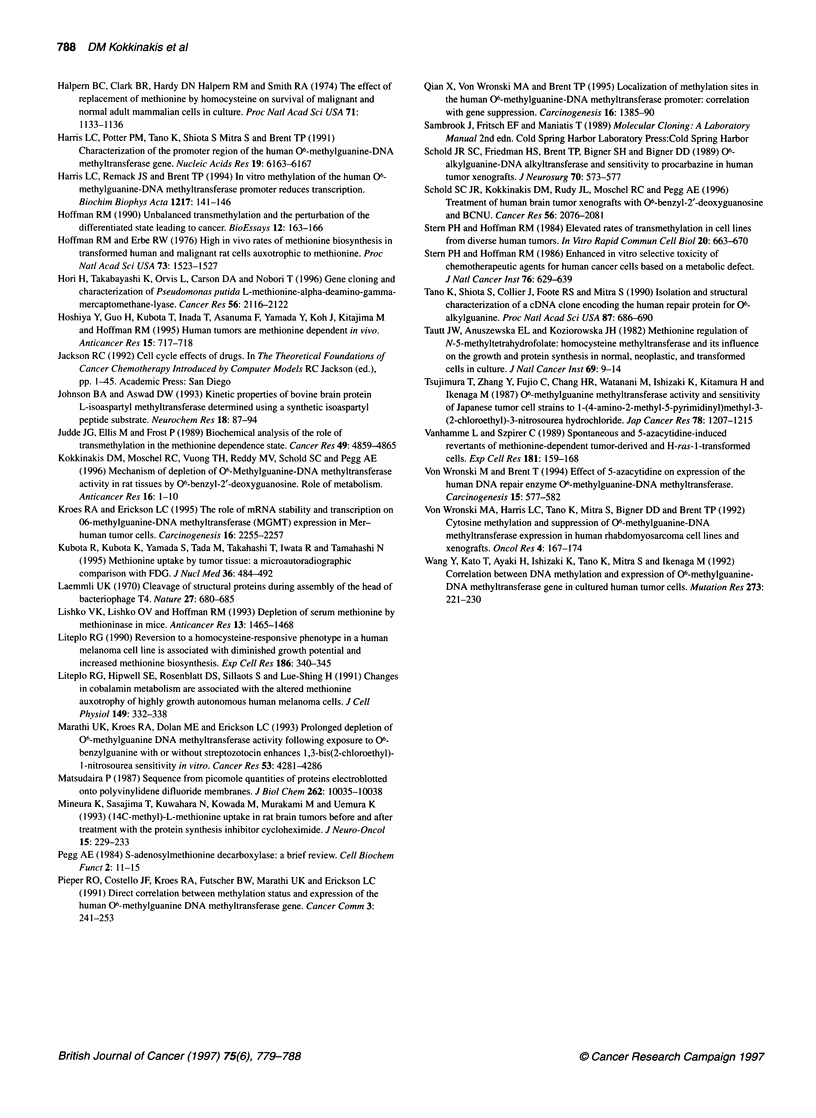# Regulation of O6-methylguanine-DNA methyltransferase by methionine in human tumour cells.

**DOI:** 10.1038/bjc.1997.141

**Published:** 1997

**Authors:** D. M. Kokkinakis, M. A. von Wronski, T. H. Vuong, T. P. Brent, S. C. Schold

**Affiliations:** Department of Neurology, The University of Texas Southwestern Medical Center, Dallas 75235-9036, USA.

## Abstract

**Images:**


British Journal of Cancer (1997) 75(6), 779–788
© 1997 Cancer Research Campaign

### Regulation of $O^6$-methylguanine-DNA methyltransferase by methionine in human tumour cells

**DM Kokkinakis[1], MA von Wronski[2], TH Vuong[1], TP Brent[2] and SC Schold Jr[1]**

[1]Department of Neurology, The University of Texas Southwestern Medical Center, 5323 Harry Hines Blvd Dallas, TX 75235–9036, USA; [2]Department of Pharmacology, The University of Tennessee, 874 Union Ave, Memphis, TN 38163, USA; [3]Department of Molecular Pharmacology, St Jude Children's Research Hospital, 332 N. Lauderdale, PO Box 318, Memphis, TN 38101 USA

**Summary** Methionine (MET)-dependent cell lines require MET to proliferate, and homocysteine (HCY) does not act as a substitute for this requirement. From six $O^6$-methylguanine-DNA methyltransferase (MGMT)-efficient (*mer*+) cell lines tested, two medulloblastomas (Daoy and D-341) and a lung non-small-cell adenocarcinoma with metastatic potential (H-1623) were most sensitive to MET deprivation, while two glioblastomas (U-138, D-263) and a small-cell lung carcinoma H-1944 were moderately to weakly dependent. Regardless of the degree of MET dependence, all of these lines down-regulated their MGMT activity within 48–72 h of transfer from MET+HCY– to MET–HCY+ media, long before the eradication of the culture. Reduction of MGMT activity was due to a decline of both MGMT mRNA and protein levels. However, the reduction was not related to the methylation status of the MGMT promoter at the *Sma*I site or the *Hpa*II sites in the body of the gene; such sites have been shown to be associated in MGMT regulation and in defining the *mer* phenotype. MET-dependent, *mer*+ tumour cells cultured in MET–HCY+ were more sensitive to BCNU ($IC_{50}$ = 5–10 μM) than those cultured in MET+HCY– ($IC_{50}$ = 45–90 μM), while MET-independent or *mer*– cell lines were unaffected. This indicates that reduction of MGMT, imposed by the absence of MET, renders *mer*+ tumour cells more susceptible to alkylating agents. The relatively selective suppression of MGMT activity in *mer*+ MET-dependent tumour cells, in combination with the inability of such cells to proliferate in the absence of MET, may lead to the development of more effective treatment strategies for *mer*+ MET-dependent tumours.

**Keywords:** methyltransferase; methionine dependence; cell cycle; $G_2$ arrest

Methionine (MET) is essential for normal growth and development of mammals. This amino acid participates in protein synthesis (Tautt et al, 1982); numerous S-adenosylmethionine-dependent transmethylation reactions (Stern and Hoffman, 1984); the formation of polyamines spermidine and spermine (Pegg 1984); synthesis of cystathionine, cysteine and other metabolites of the transulphuration pathway; the supply of homocysteine (HCY), which is needed for metabolism of intracellular folates; and the catabolism of choline (Finkelstein, 1990). With very few exceptions, normal cells can use HCY in place of MET to support all of the above reactions (Hoffman, 1990; Guo et al, 1993*a*). In contrast to normal cells, a large number of cultured tumour cells and about 25% of fresh human tumours grown in histocultures cannot effectively use HCY in place of MET, and such cells or tumours are classified as MET-dependent (Guo et al, 1993*a*,*b*). There is substantial evidence that MET dependence occurs more frequently in metastatic tumour cells (Breillout et al, 1987, 1990; Liteplo, 1990), although reversion of dependence is not necessarily linked to loss of metastatic potential (Vanhamme and Szpirer, 1989). The biochemical basis for MET dependency is not yet fully understood. MET-dependent tumour cells appear to synthesize MET from HCY by an active MET synthase, but at levels not adequate to both sustain growth and meet their high transmethylation requirements (Judde et al, 1989). The most likely biochemical defect leading to MET dependency is thought to be related to the synthesis and availability of methylcobalamine, which is directly involved in the transfer of methyl groups from 5-methyltetrahydrofolate to HCY (Liteplo et al, 1991; Fiskerstrand et al, 1994).

The defect in the use of HCY has been extensively investigated to induce selective killing of tumours while sparing normal tissues. Lack of MET results in a reversible blockage of rapidly proliferating tumour cells in the late S and $G_2$ phases of the cell cycle (Stern and Hoffman, 1986; Guo et al, 1993*a*). In general, cells arrested in S, $G_2$ or M are not only susceptible to spontaneous death, unlike cells arrested in $G_1$, but they are also supersensitive to various chemotherapeutic drugs, such as doxorubicin, which blocks and kills in $G_2$ (Stern and Hoffman, 1986). MET-depleting diets in combination with chemotherapy suppress metastasis of Yoshida sarcoma and rhabdomyosarcoma tumours in animals (Breillout et al, 1987; Goseki et al, 1992). Potentiation of many drugs, some of which act by damaging cellular DNA, indicates that MET deprivation may compromise DNA repair ability in addition to causing premitotic cell cycle blocks and cell death in tumours. The modulation of resistance of tumour cells to chemotherapy may encompass a large number of mechanisms including those involved in the processing of the agent (metabolism, conjugation, etc.), its transport into or its elimination from the target cell, or the reversal or repair of the damage induced by such agents. A systematic study of the modulation of such mechanisms by deprivation of MET will allow the deployment of the full potential of the MET-dependence phenotype in the treatment of several tumours. In that context, we examined the effect of MET depletion on the ability of tumour cells to repair $O^6$-alkylguanine adducts by $O^6$-methylguanine-DNA methyltransferase (MGMT).

*Received 16 January 1996*
*Revised 17 September 1996*
*Accepted 24 September 1996*

*Correspondence to:* DM Kokkinakis

Such adducts are major contributors to the toxicity of alkylating anti-tumour agents, such as procarbazine, temozolomide, carmustine and related nitrosoureas commonly used for the treatment of several tumours (Schold et al, 1989; Dunn et al, 1991; Egyhazi et al, 1991; Brent et al, 1993; Marathi et al, 1993).

### METHODS

### Cell culture

NIH/3T3 from American Type Culture Collection (ATCC Rockville, MD, USA) was used as a negative control for MET dependence. SWB-40 and U-87, both *mer*⁻ human anaplastic glioma cell lines, were used to determine the effect of MET depletion on BCNU resistance by mechanisms other than those that are MGMT mediated. The effect of MET deprivation on cell growth and MGMT levels was examined in Daoy and D-341 (human medulloblastomas), U-138 and D-263 (human glioblastomas), H-1994 (a human small-cell lung carcinoma) and H-1623 (a human non-small-cell lung adenocarcinoma with metastatic ability). The brain tumour cell lines were obtained either from ATCC (U-138, Daoy, U-87) or were donated by Dr H Friedman (Department of Pediatrics, Duke University) (D-341, D-263). The lung tumour cell lines were donated by Dr A Gazdar (Department of Pathology, UT Southwestern) and were adapted to our medium. All cells were maintained in culture in Eagle's minimum essential medium (Gibco) supplemented with lysine, valine and leucine (100 μM each) and with 10% dialysed MET-free fetal bovine serum. The medium was also supplemented with non-essential amino acids (1:100 dilution of stock from Gibco), 1 mM sodium pyruvate, sodium bicarbonate, 6.0 μM α-hydroxycobalamin, 100 μM folic acid, 0.2 mg $ml^{-1}$ gentamicin and either 100 μM L-MET ($MET^+HCY^-$) or 200 μM D,L-HCY thiolactone ($MET^-HCY^+$). Cells were plated in $MET^+HCY^-$ medium and were allowed to attach and grow until they reached 50% confluency ($6 \times 10^6$ per flask). Detaching cells ascertained to be dead by trypan blue exclusion were removed by changing the medium every 48 h. Cells were then either trypsinized and reseeded in the same medium or washed with PBS and supplied with the $MET^-HCY^+$ medium. Because of extensive death in this medium, it was necessary to remove detaching dead cells on a daily basis. Cells from both media were harvested at time intervals indicated and used to measure cell cycle status and MGMT activity. Cells from either cultures ($5 \times 10^7$) were also harvested, washed with PBS and immediately frozen as a source of protein, DNA and RNA. All cells collected were live (>95%) as determined by trypan blue exclusion.

### Staining for DNA analysis

Trypsinized cells were washed with PBS three times and spread on slides with a cytospin centrifuge at 500 r.p.m. for 1 min. Slides were dipped in Fix-Rite for 1 min, washed with water and 1 N hydrochloric acid and incubated in preheated hydrochloric acid at 60°C for 15 min. The slides were rinsed with water, stained with Schiff's reagent for 45 min at room temperature, treated with two successive changes of freshly prepared sulphurous acid, rinsed well and dehydrated. Finally, slides were cover-slipped for DNA analysis. DNA content was measured by image analysis using a V-I 470 Optronics camera and Bio-Quant system IV software. Three to four hundred cells were measured in each slide and the distribution, based on DNA content, was determined with Lotus 123 software. A control of normal lymphocytes was used to assign DNA content values. MET-dependent cell cycle blocks (MDCCB) were determined according to Guo et al (1993*a*) using the equation:

$$\text{MDCCB} = \text{per cent of } G_1 \text{ cells (in } MET^-HCY^+\text{)/per cent of } G_1 \text{ cells (in } MET^+HCY^-\text{)}$$

where $G_1$ is the number of cells in this cell cycle phase and represented by the cells under the first peak of a DNA-distribution graph.

MDCCB numbers below one indicate a $G_2$ block, while above one are consistent with $G_1$ blocks.

### MGMT activity measurements

#### *Substrate preparation*

The [$^3$H]DNA substrate for measurements of MGMT activity was synthesized as follows: One millicurie of ethanolic solution of *N*-[$^3$H]methyl-*N*-nitrosourea (MNU), having specific activity 18.8 Ci $mmol^{-1}$ (Amersham), was concentrated to 0.2 ml with a nitrogen stream and mixed with 1 ml of DNA solution (5 mg) in 0.02 M 2-amino-2-methyl-1, 3-propanediol at pH 10. The solution was incubated for 30 min at 37°C in a water bath and another 30 min at room temperature. DNA was precipitated by adding 10% (v/v) 2.5 M sodium acetate and three volumes of cold ethanol and allowed to stand for 18 h at – 20°C. Precipitated DNA was washed with 70% ethanol (three times) and 100% ethanol (two times) and redissolved in 3 ml of 10 mM Tris, 1 mM EDTA buffer at pH 7.4.

#### *Substrate characteristics*

The [$^3$H]DNA contained 29.8 μCi of radioactivity (alkylation efficiency 3%) which was distributed as 3-methyladenine, 3.5%; 7-MeG, 79.6%; 1-methyladenine, 0.6%; $O^6$-MeG, 9.0%; and other adducts, 7.3%. The specific activities of 7-MeG and $O^6$-MeG were calculated to be 15.8 ± 0.8 and 16.5 ± 0.5 Ci $mmol^{-1}$ respectively. For all practical purposes, these were considered equal, although the specific activity of the adducts was only 86% of that reported for the MNU. Accordingly, the ratio of $O^6$-MeG to 7-MeG was 0.113.

#### *Cell extracts*

Cells were pelleted at 800 r.p.m. and suspended in five volumes of 100 mM Tris.HCl containing 0.1 mM EDTA and 2 mM DTT with the pH adjusted to 7.8. Cell suspensions were freeze–thawed three times using liquid nitrogen and sonicated for 10 s (three times) using 70% maximum output. A small aliquot was removed for DNA determination. Cell debris was removed by spinning at 18 000 *g* for 10 min at 0°C and the supernatant removed and frozen in liquid nitrogen until used. Protein was determined by the method of Bradford 1976).

#### *MGMT assay*

[$^3$H]DNA dissolved in 100 mM Tris, 0.1 mM EDTA, 2 mM DTT at pH 7.8 and containing 60 fmol of $O^6$-MeG (total d.p.m. $24 \times 10^3$) was incubated with 0–500 μg of protein (depending on suspected MGMT activity) for 1 h at 37°C in a final volume of 500 μl. The reaction was quenched with 0.1 ml of 1 N HCI, and samples were incubated for an additional 45 min at 70°C. Samples were cooled on ice for 1 h, centrifuged at 14 000 *g* for 5 min, the supernatant removed and neutralized with sodium bicarbonate, and dried by lyophilization. Lyophilized samples were dissolved in 0.12 ml

© Cancer Research Campaign 1997

0.1 M HCl, spun at 14 000 *g* and analysed by HPLC using a Supelcosil-C18DB analytical column (Supelco). Samples were eluted at a flow rate of 1.5 ml min$^{-1}$ with 2% acetonitrile in 0.1 M phosphate buffer pH 3.5 (0–5 min) followed by a gradient of 1% acetonitrile per min (5–15 min). Radioactivity was monitored by fraction collection and scintillation counting. The 7-MeG and $O^6$-MeG were eluted at 4.5 and 13 min respectively. The ratio of radioactivity under the $O^6$-MeG over that of 7-MeG from four samples of varying protein concentration was derived and plotted against the amount of protein. The intercept of the central linear response of the curve (between ratios 0.9 and 0.3) with the *x*-axis marks the amount of protein needed to remove 60 fmol of $O^6$-MeG from DNA. The assay is sensitive enough to detect MGMT levels as low as 5 fmol mg$^{-1}$ protein with an error of less than 10%.

### SDS-polyacrylamide gel electrophoresis and immunoblotting

Proteins were resolved in a Bio-Rad (Richmond, CA, USA) minigel apparatus at 200 V for 45 min on 0.75 mm SDS-PAGE slab gels using the method of Laemmli (1970). The gels were calibrated with Bio-Rad low molecular weight standards. Proteins were transferred onto PVDF membrane (Immobilon-P, Millipore, Bedford, MA, USA) by the method of Matsudaira (1987) using a Bio-Rad Mini trans-Blot cell for 2 h at 140 mA. Blots were blocked with 5% bovine serum albumin (BSA) in 20 mM Tris pH 8.2 with 0.9% sodium chloride and were probed for 2 h with mouse monoclonal antibody MT3.1 specific for human MGMT in a buffer containing 20 mM Tris pH 8.2 0.1% BSA, 0.9% sodium chloride, 1% normal goat serum and 5% concentrated gelatin solution (Amersham). Antibody binding was visualized with Amersham's gold-labelled secondary antibody and silver enhancement using Auroprobe and IntenSE reagents (Amersham), according to manufacturer's instructions. The intensities of bands were quantitated in a photographic positive by whole band analysis on a Bio-Image Visage 110 analytical imaging instrument (Millipore).

### DNA probes

MGMT cDNA was derived by polymerase chain reaction (PCR) of the cloned insert in the plasmid pKT100 (Tano et al, 1990) using #1201 and #1211 sequencing primers (New England Biolabs, Beverly, MA, USA) with a GeneAmp kit (Perkin Elmer Cetus, Norwalk, CT, USA) using the starting parameters recommended by the manufacture. Twenty-five cycles of amplification were carried out, with each cycle consisting of 30 s at 94°C, 1 min at 45°C and 1 min at 72°C. A 772-bp sequence from the MGMT promoter was obtained by restriction of the plasmid pKT200 (Harris et al, 1991) with Sstl (Gibco-BRL, Gaithersburg, MD, USA) and Pstl (New England Biolabs) and excision of the fragment from low-melting point agarose gel.

### Northern Analysis

Total RNA was prepared from frozen cells by lysing them in RNAzol (Tel-Test, Friendswood, TX, USA) according to the manufacturer's protocol. RNA (10 μg) was size fractionated in 1% agarose gels containing 2.2 M formaldehyde (Fisher, Fair Lawn, NJ, USA) (Sambrook et al, 1989) and transferred to nylon membranes (Duralon-UV, Stratagene, La Jolla, CA, USA) by capillary blotting. RNA was covalently attached with UV and prehybridized for 4 h at 42°C in 50% formamide, 5 × SSPE (0.18 M sodium chloride, 0.01 M sodium phosphate, 1 mM EDTA), 5 × Denhardt's solution (Sambrook et al, 1989), 7.5% dextran sulphate, 1.5% sodium dodecyl sulphate (SDS), and 200 μg ml$^{-1}$ of sheared salmon sperm DNA. Hybridization was conducted with $^{32}$P-labelled MGMT cDNA probe for 20 h at 42°C. Unbound probe was removed by washing membranes twice with 200 ml of 2 × SSC (0.015 M sodium chloride, 0.0015 M sodium citrate) for 15 min at room temperature, followed by washing twice with 200 ml of 0.1 × SSC, 0.1% SDS for 15 min at 65°C. Membranes were exposed to X-Omat AR Kodak film at –70°C, and RNA was quantitated. Membranes were reprobed with GAPDH cDNA (Clontech, Palo Alto, CA, USA) to control for equal loading and transfer. After this hybridization, membranes were washed as before except that the final step was performed at 68°C instead of 65°C.

### Analysis of MGMT gene methylation

Ten micrograms of genomic DNA from each cell line, isolated according to protocol 1 of Sambrook et al (1989) was restricted with 5 U μg$^{-1}$ of *Eco*RI and *Hpa*II (New England Biolabs) for 4–6 h and then restricted again with the same amount of enzyme overnight. Electrophoresis of the samples in a 0.7% agarose gel containing 0.5 × TBE at 22 V for 18 h was followed by alkaline denaturation and neutralization, according to Sambrook et al (1989), and capillary blotting onto a nylon membrane as in Northern analysis and covalent linking of the DNA using UV light. The blot was hybridized with a $^{32}$P-labelled MGMT cDNA probe and washed as described for Northern analysis before autoradiography for 2 days at –70°C with intensifying screens.

### Analysis of MGMT promoter methylation

Methylation of the 5′-untranslated region of the MGMT gene was investigated by Southern analysis using the methylation-sensitive restriction enzyme *Sma*I (Promega, Madison, WI, USA). Genomic DNA was restricted with 5 U μg$^{-1}$ of enzyme at 37°C (25°C for *Sma*I) for 4–6 h and then restricted again with the same amount of enzyme overnight. Southern analysis was performed using the 772-bp probe from the 5′-untranslated region.

### Cytotoxicity assays

The cytotoxicity of BCNU on cultured cells in MET$^+$HCY$^-$ and MET$^-$HCY$^+$ was measured by a modification of the method of Branch et al (1993) as follows: MET-dependent cells were seeded and cultured in MET$^+$HCY$^-$ in 35-mm Petri dishes until they were nearly confluent. The cells were then transferred in MET$^-$HCY$^+$ and cultured for an additional 4–6 days at which time cell numbers were reduced because of cell death and inhibition of mitosis. Dead cells were removed and medium was replaced on a daily basis. Following this period in MET$^-$HCY$^+$ medium, remaining live cells were washed with PBS and then treated with various concentrations of BCNU in PBS for 1 h at 37°C while they were still attached. Subsequently, the' BCNU was removed, replaced with MET$^+$HCY$^-$ medium, and cultures were incubated for an additional 4 days at 37°C in 6% carbon dioxide. At that time cells were trypsinized and counted using a Coulter-Counter (Coulter

© Cancer Research Campaign 1997

Electronics, Hialeah, Fl, USA). The concentration of the drug that halves the growth rate of the tumour cells ($IC_{50}$) was determined from plots of BCNU concentrations vs the per cent change in cell numbers compared with untreated controls within a 4-day interval from treatment (Jackson, 1992). The $IC_{50}$ of BCNU on MET-dependent cells cultured in $MET^+HCY^-$ or MET-independent cells cultured in either media was also determined by the same method. With such cultures, however, cell numbers originally seeded were adjusted to yield cell densities that were similar to those of $MET^-HCY^+$ cultures at the time of treatment with BCNU.

### RESULTS

### Inhibition of cell proliferation by methionine withdrawal

Doubling times for MET-dependent, *mer*+ Daoy, D-341, U-138, D-263, H-1944 and H-1623 in $MET^+HCY^-$ were estimated from cell counting as 26, 34, 45, 42, 56 and 46 h respectively. Doubling times for *mer*− U-87 and SWB-40 tumour cells and NIH-3T3 were 45, 32 and 26 h respectively. Figure 1 shows that, with the exception of U-87 which continued to proliferate in $MET^-HCY^+$, the rest of the tumour lines tested were MET dependent. This was best demonstrated with Daoy, H-1623 and D-341 which were nearly eliminated 6 days following their transfer from the $MET^+HCY^-$ to $MET^-HCY^+$ medium. A less rapid or extensive reduction in cell populations within the same time period was also observed in U-138, SWB-40, D-263 and H-1944 cultures in $MET^-HCY^+$. After this initial cell loss, further decline of populations was observed in all tumour cell lines, although at a slower rate. The biphasic rates of cell loss in $MET^-HCY^+$ cultures was indicative of at least two different mechanisms of toxicity. Examination of cell cycle kinetics indicated the accumulation in late S and $G_2$ at day 2 from transfer to MET-deficient media (Figure 2), which was characteristic of a $G_2$ cell cycle block. Cell cycle kinetic analysis for the Daoy, U-138, H-1632 and D-341 is shown in Table 1. MET-dependent $G_2$ cell cycle blocks (MDCCB<0.8) were observed in all of these four lines, but they were not detectable in H-1944, SWB-40 and D-263, which were moderately to weakly dependent on MET. A $G_2$ block was imposed earlier in H-1623, D-341 and Daoy than in U-138 and was generally followed by a massive detachment and death of cells (Figure 1) and a concomitant increase of the MDCCB (Table 1). Following the $G_2$ block and elimination of $G_2$-arrested cells, the mitotic index declined rapidly by more than an order of magnitude below the control value as the MDCCB number increased above one, indicating that surviving cells did not cycle and were most probably checked at $G_1$. MDCCB fluctuations consistent with $G_2$ or $G_1$ blocks were not found in NIH-3T3 or U-87 which were not MET dependent and proliferated in $MET^-HCY^+$. Cell cultures of MET-dependent cells tested here could be rescued, even after elimination of more than 95% of the original cell population, by replenishing MET in the medium. Karyotypic analysis before MET depletion, and also 48 h after the repletion of MET in a Daoy culture deprived of MET for ten days, indicated that no additional chromosomal aberrations or selection of a resistant cell population were introduced by this treatment. In all cell lines tested, repletion of MET in the medium resulted in the recovery of both cell proliferation capacity and base line MGMT activity. In Daoy, recovery of the MGMT activity was not complete 48 h (65% of the base line) from repletion of MET following a 10-day culture period in HCY (DM Kokkinakis, unpublished observations). At that time cells had recovered their ability to divide and proliferated with a doubling time of 22 h.

**Figure 1** Effect of replacement of methionine with homocysteine on cultures of the Daoy (o), D-341 (●), U-138 (▽), D-263 (▼), H-1944 (△), H-1623 (▲), U-87 (□), SWB-40 (■) and NIH-3T3 (◆). Cells growing exponentially in $MET^+HCY^-$ were washed with PBS two times and seeded in $MET^-HCY^+$ medium. Cultures were trypsinized, and live cells, as determined by trypan blue exclusion, were counted

### Reduction of MGMT activity in tumour cells by methionine depletion

The native MGMT activities of the cell lines tested here are shown in Table 2. These activities were not affected by serum withdrawal and the associated inhibition of growth for up to 72 h, and they were not dependent on the state of confluency of the culture (data not shown). A cell cycle-dependent regulation of MGMT activity, previously suggested (Dunn et al, 1986) by serum starvation synchronization, was also not evident in these lines. However, MGMT activities were dependent on the presence of MET in the culture medium (Table 2). With the exception of NIH-3T3 cells, which were not MET dependent, MGMT activity declined with a half-life ranging from 24 to 48 h after cells were transferred to $MET^-HCY^+$ media and reached a nadir of 25–40 fmol $mg^{-1}$ protein in all the lines tested. Levels of MGMT activity remained low in MET-dependent cells as long as such cells remained alive in the $MET^-HCY^+$ medium. In Daoy, the decline in the MGMT activity in $MET^-HCY^+$ was due to the reduction of MGMT protein (Figure 3). The amount of MGMT protein determined by densitometric scanning 6 days after culturing in $MET^-HCY^+$ was reduced eightfold, which was similar to the decline of MGMT activity. Northern analysis (Figure 4) indicated that the reduction of the protein and activity was reflected by a similar reduction of mRNA without notable reduction of mRNA for GAPDH, another 'housekeeping' protein. Reduction of the MGMT mRNA was notable 48 h after

© Cancer Research Campaign 1997

**Figure 2** Effect of replacement of methionine with homocysteine on the distribution of cell populations of Daoy in $G_1$, S and $G_2$ compartments. Cell cycle distribution in $MET^+HCY^-$ (**A**) and $MET^-HCY^+$ after 2 (**B**), 4 (**C**) and 6 (**D**) days of culture. A $G_2$ block results in a shift of distribution from $G_1$ to $G_2$ as early as day 2

transfer to $MET^-HCY^+$ medium and further declined to levels that were barely detectable a week after MET deprivation. The above suggests that MET deprivation affects MGMT transcription or mRNA stability and probably not MGMT protein translation or stability. Down-regulation of MGMT expression by MET deprivation could be theoretically related to changes in methylation of cytosine in key positions of the MGMT gene and promoter because of the decline of the capacity of cells to transmethylate when cultured in a HCY medium. Recent studies have shown that cytosine methylation influences MGMT gene expression (von Wronski et al, 1992; Wang et al, 1992; Costello et al, 1994). Methylation of the MGMT promoter is associated with loss of gene expression, while methylation of the gene itself appears to enhance its expression. Specifically methylation in the *Sma*I restriction site (–69) of the MGMT promoter has been found only in *mer*⁻ tumour cell lines (von Wronski et al, 1992). On the other hand, 5-azacytidine induced methylation of the body of the gene at *Hpa*II-sensitive sites results in a substantial increase of the MGMT activity in some tumour cell lines (von Wronski and Brent, 1994). Surprisingly, decline of mRNA induced by MET withdrawal was not associated with changes in the methylation status of the MGMT promoter or the body of the gene at the *Sma*I and *Hpa*II sites respectively (Figure 5). In Daoy, the *Sma*I site at –69 was entirely unmethylated in both the control ($MET^+HCY^-$) and down-regulated cells ($MET^-HCY^+$), which is consistent with the observation that this line remains *mer*⁺ even in the absence of MET. Lack of an effect of MET deprivation on the methylation of the *Sma*I and *Hpa*II sites was also observed in the *mer*⁺, MET-dependent U-138 (data not shown).

### Potentiation of the toxicity of nitrosoureas by methionine depletion

The *mer*⁺ Daoy line was resistant to BCNU in $MET^+HCY^-$ with an $IC_{50}$ of 45 μM. Such resistance was compromised and the $IC_{50}$ was

**Table 1** Variation of MDCCB[a] and mitotic activity after transfer of MET-dependent tumour cell cultures to MET⁻HCY⁺ medium

| Cell line | Time (days) | MDCCB | Mitotic index % of control[b] |
|---|---|---|---|
| DAOY | 1 | 0.70 ± 0.10[c] | 51 ± 6[c] |
| | 2 | 0.45 ± 0.11 | 24 ± 4 |
| | 3 | 0.89 ± 0.08 | 12 ± 4 |
| | 4 | 1.10 ± 0.06 | 3 ± 2 |
| | 5 | 1.62 ± 0.11 | 0 |
| | 6 | 1.68 ± 0.13 | 0 |
| U-138 | 2 | 0.85 ± 0.09 | 38 ± 11 |
| | 4 | 0.61 ± 0.08 | 21 ± 7 |
| | 6 | 0.99 ± 0.12 | 6 ± 3 |
| | 8 | 1.05 ± 0.09 | 0 |
| | 10 | 1.45 ± 0.09 | 0 |
| H-1623 | 1 | 0.42 ± 0.06 | 27 ± 5 |
| | 2 | 0.88 ± 0.04 | 13 ± 3 |
| | 4 | 1.35 ± 0.12 | 5 ± 2 |
| | 6 | 1.57 ± 0.11 | 0 |
| D-341 | 1 | 0.66 ± 0.08 | 49 ± 11 |
| | 2 | 0.45 ± 0.06 | 12 ± 4 |
| | 4 | 0.89 ± 0.09 | 0 |
| | 6 | 1.06 ± 0.11 | 0 |
| | 8 | 1.31 ± 0.12 | 0 |

[a] MDCCB, MET-dependent cell cycle blocks. Numbers < 1.0 show accumulation of cells in $G_2$, while >1 demonstrate a $G_1$ block. [b] Mitotic indices (mitosis per 1000 cells) for Daoy, U-138, H-1623 and D-341 in MET⁺HCY⁻ were 49, 39, 52 and 67 respectively. [c] Mean of three experiments ± standard deviation.

**Table 2** Time dependence of MGMT activity in MET⁻HCY⁺ cultures

| Cell line | Time[a] | MGMT[b] | Cell line | Time[a] | MGMT[b] |
|---|---|---|---|---|---|
| DAOY | 0 | 383 ± 41[c] | H-1623 | 0 | 880 ± 51 |
| | 2 | 85 ± 9 | | 2 | 326 ± 62 |
| | 4 | 61 ± 5 | | 4 | 132 ± 9 |
| | 6 | 32 ± 4 | | 6 | 51 ± 13 |
| | 8 | 35 ± 3 | | 8 | 41 ± 12 |
| | 10 | 33 ± 5 | | | |
| U-138 | 0 | 384 ± 35 | H-1944 | 0 | 328 ± 11 |
| | 2 | 140 ± 13 | | 2 | 263 ± 28 |
| | 4 | 85 ± 9 | | 4 | 143 ± 17 |
| | 6 | 33 ± 4 | | 6 | 75 ± 5 |
| | 8 | 21 ± 4 | | 8 | 48 ± 5 |
| | 10 | 25 ± 3 | | 10 | 42 ± 5 |
| D-341 | 0 | 361 ± 18 | NIH3T3 | 0 | 236 ± 33 |
| | 2 | 139 ± 15 | | 2 | 287 ± 18 |
| | 4 | 63 ± 8 | | 4 | 285 ± 32 |
| | 6 | 35 ± 4 | | 6 | 291 ± 43 |
| | 8 | 33 ± 4 | | 8 | 311 ± 58 |

[a]Days after replacing MET⁺HCY⁻ with MET⁻HCY⁺ medium. [b]f mol mg$^{-1}$ protein. [c]Standard deviation from three determinations.

**Figure 3** Immunoblot analysis of MGMT protein in Daoy cell line cultured in either MET⁺HCY⁻ (M) or in MET⁻HCY⁺ (H) media. Twenty micrograms of extract protein was electrophoresed in a 12% SDS-polyacrylamide gel. Proteins were electroblotted onto a PVDF membrane which was probed with monoclonal antibody MT3. 1 specific for human MGMT. MGMT in the human leukaemic lymphoblast line CEM-CCRF is shown as control reduced to approximately 5 μM when cells were cultured in MET⁻HCY⁺ for 4 days before the exposure to BCNU (Figure 6A). In comparison, the resistance of the *mer⁻*, MET-dependent SWB-40 to BCNU was not affected by MET depletion (Figure 6B). The similar response of *mer⁺* Daoy and *mer⁻* SWB-40 to BCNU in MET⁻HCY⁺ and the demonstrated association between BCNU sensitivity and MGMT activity in Daoy suggests that this line owes its resistance to alkylating agents to the high levels of MGMT reserves. On the other hand, the marginal effect of MET deprivation on the resistance of SWB-40 to BCNU suggests that down-regulation of MGMT activity is probably the major pathway for the loss of resistance to BCNU and to related alkylating agents in association with the MET-dependence phenotype. An extensive correlation between the down-regulation of MGMT activity by methionine deprivation and the sensitization to BCNU was demonstrated with several *mer⁺*, MET-dependent cell lines (Table 3).

### DISCUSSION

Tumours have high MET requirements because of accelerated protein synthesis and transmethylation reactions to yield S-adenosylmethionine, serine, sarcosine, glycine and various phospholipids (Mineura et al, 1993; Kubota et al, 1995). Twenty-five per cent of human tumours are estimated to be absolutely dependent of MET and cannot use HCY to either proliferate or survive (Guo, 1993). A greater percentage is expected to be moderately to weakly dependent on MET and to respond, to some extent, to MET deprivation (Hoshiya et al, 1995). In this regard

 © Cancer Research Campaign 1997

**Figure 4** Northern analysis of MGMT mRNA expression in Daoy cultured in either MET+HCY− (M) or in MET−HCY+ (H). Twenty micrograms of total RNA from a MET+HCY− culture and from cultures deprived of MET (MET−HCY+) for 2, 4 or 7/8 days respectively were subjected to electrophoresis, transferred to a nylon membrane and probed with $^{32}P$-labelled human MGMT cDNA (top). Reprobing the membrane with $^{32}P$-labelled GAPDH cDNA (bottom) demonstrates similar loading for all samples. Lanes 1–4 and 5–8 represent duplicate Northern analyses

**Figure 5** Southern analysis of methylation sensitive restriction sites in the MGMT gene in Daoy cultured in either MET+HCY− (M) or in MET−HCY+ (H). Ten micrograms of DNA from each culture was either restricted with *Sac*I and *Sma*I and probed with the 772-bp MGMT promoter fragment (**A**) or with *Eco*RI and *Hpa*II and probed with MGMT cDNA (**B**). MET deprivation has no effect on cystosine methylation in either the MGMT promoter (*Sma*I, –69 site) or in the body of the gene

**Figure 6** Effect of methionine depletion on the sensitivity of the *mer*−SWB-40 (**A**) and the *mer*+ Daoy (**B**) cell lines to BCNU. Cells previously cultured in MET+HCY− (o) or in MET−HCY+ (●) for 4 (Daoy) or 6 (SWB-40) days were exposed to BCNU for 1 h at 37°C and subsequently cultured in MET+HCY− medium. Live cell populations were determined at day 4 after BCNU treatment in expanding cultures and plotted against BCNU concentrations. Cell numbers were compared with those of their respective control (no BCNU) to determine BCNU-induced growth inhibition and death

© Cancer Research Campaign 1997

**Table 3** Sensitization of MET-dependent tumour cells to BCNU by MET withdrawal

| Cell line | Remaining MGMT[a] | $IC_{50}$ (MET)[b] | $IC_{50}$ (HCY) |
|---|---|---|---|
| Daoy | 8.6 | 45 | 5 |
| U-138 | 6.5 | 50 | 10 |
| D-341 | 9.1 | 65 | 10 |
| H-1623 | 4.6 | 90 | 10 |
| H-1944 | 12.8 | 65 | 10 |
| U-87[c] | ND | 15 | 15 |
| SW-40[c] | ND | 10 | 15 |
| NIH-3T3[d] | 131.8 | 35 | 40 |

[a]Per cent MGMT activity remaining after exhaustive deprivation of exogenous MET. [b]Concentration (µM) of BCNU needed to reduce relative survival by 50%, 4 days following 1-h incubation with the drug. [c]*mer*⁻ lines, not detectable (ND) MGMT. [d]Control *mer*, MET-independent cell line.

MET deprivation is an interesting approach to therapy of a variety of tumours assuming that reduction of plasma methionine below the threshold needed to suppress cell proliferation in the tumour can be achieved. As the exogenous MET requirement of MET-dependent tumour cell lines varies (Halpern et al, 1974; Hoffman and Erbe 1976; Breillout et al, 1987), the behaviour of tumours in vivo, where MET cannot be completely eliminated, is difficult to predict. Prolonged reduction of plasma MET can be non-toxic in animals if HCY is supplemented to supply normal tissues with a MET precursor. Thus, an eighty per cent reduction of plasma MET has been obtained in athymic mice fed a MET-free diet. Further reduction (approximately 95%) can be achieved with the use of L-methionine-α-deamino-γ-mercaptomethane lyase, also known as methioninase (Lishko et al, 1993), an enzyme that has been recently cloned and mass produced (Hori et al, 1996). A combination of MET-depleting diets and methioninase could theoretically cause a decrease in plasma MET to less than 5 µM, which is the threshold for supporting cell proliferation in all MET-dependent tumour cell cultures examined (DM Kokkinakis, unpublished observations).

Three out of eight tumours tested here are strongly dependent on MET and are rapidly and nearly eradicated, mainly as the result of $G_2$ blocks induced by MET deprivation. The rest of the tumours tested, including all gliomas, resisted MET starvation (in the presence of HCY) and were able to survive for several weeks and resume growth when MET was repleated. Prolonged survival, possibly as the result of $G_1$ cell cycle blocks of MET-dependent tumour cells is expected to be a major problem in any effort to eradicate tumours based entirely on MET-depleting regimens. In this regard, although MET depletion may be useful in replacing toxic chemotherapeutic treatments, its greater application could be in conjunction with currently used chemotherapeutic agents, as previously suggested (Stern and Hoffman, 1986; Breillout et al, 1987; Goseki et al, 1992).

A major mechanism of resistance of cells to genotoxic injury, particularly to the formation of the toxic and mutagenic $O^6$-alkylguanine DNA adducts and to subsequent lethal DNA cross-links, is mediated by the DNA repair protein MGMT. This protein, which reverses formation of $O^6$-alkylguanine adducts including those with the potential to react with DNA bases of the opposite strand and form cross-links, is abundant in more than eighty per cent of the human tumours, rendering them resistant to a variety of genotoxic alkylating chemotherapeutic agents (Day et al, 1980; Tsujimura et al, 1987; Dolan et al, 1990). In many cases, levels of MGMT activity in tumour tissue are well above that of the normal surrounding tissue or of other vital tissues (Gerson et al, 1985). This is believed to be the main reason for the poor therapeutic index of many chemotherapeutic genotoxic drugs that alkylate the $O^6$-position of guanine used in chemotherapy. Significant increase in the efficacy of alkylating chemotherapeutic drugs, such as BCNU, against MGMT-positive tumours has been obtained with the prior depletion of MGMT activity both in tumour and in normal tissue by $O^6$-benzylguanine and its analogues (Dolan et al, 1993; Schold et al, 1996). Depletion of MGMT in animals treated with $O^6$-benzylguanine analogues is effective provided that the inhibitor and its active metabolites are present in adequate concentrations to sustain destruction of newly synthesized MGMT (Kokkinakis et al, 1996). As MGMT inhibitors inactivate only existing protein and have no effect on the transcription or translation of the stable MGMT message, MGMT activity appears immediately after clearance of the inhibitor owing to continuous translation of persisting MGMT mRNA. An even greater increase in the efficacy of BCNU, and similar genotoxic drugs that kill primarily because of $O^6$-alkylguanine adducts, can be achieved by depleting both the MGMT protein and its mRNA only in the tumour while leaving normal tissues unaffected. Theoretically, this can be accomplished in tumours that possess both the MET dependent and *mer*⁺ phenotypes by MET-depleting regimens.

Selective depletion of both the MGMT protein and its mRNA can be imposed on tumour cells by MET deprivation. Depletion of the activity and most probably of the protein in Daoy, U-138, D-341 and H-1623 follows first-order kinetics with half-lives varying between 36 and 48 h. In H-1994 and D-263, which are weakly dependent on MET, a lag period of approximately 48 h precedes such decline of activity. The detection of MGMT mRNA in Daoy, 8 days after MET withdrawal, suggests that transcription of the MGMT gene is not completely silenced in a MET-depleted state. Persistence of MGMT mRNA for that length of time without new synthesis is unlikely in spite of the reported stability of the MGMT message in several tumour cells lines (Kroes and Erickson, 1995). Low levels of MGMT found in $MET^-HCY^+$ for several days after reaching a nadir further demonstrate the slow rate of basal transcription of this gene by cells that are apparently blocked in $G_1$. In the simplest scenario, MET withdrawal inhibits transcription but has no effect on the stability of mRNA and protein. A gradually declining MGMT activity therefore reflects the difference between degradation of the protein with a half-life of approximately 20 h (Brent et al, 1991) and translation of pre-existing mRNA, which is also on a decline, with a half-life of approximately 12 h (Kroes and Erickson, 1995). Resynthesis of protein, mainly from translation of pre-existing mRNA, may influence the kinetics of MGMT decline by increasing the apparent half-lives of the MGMT activity from the expected 20 h up to 48 h depending on the tumour line. A lag period in the decline of MGMT activity observed in H-1944 is probably the result of the ability of this cell line to use HCY and maintain higher levels of endogenous MET than Daoy (data not shown). The mechanism of down-regulation of MGMT activity by MET deprivation could potentially involve changes in the methylation of the gene. Such changes, particularly at the promoter region of the MGMT gene, have already been recognized as having an intimate association with cellular levels in MGMT activity. Thus, a direct correlation

 © Cancer Research Campaign 1997

between methylation in the body of the gene and MGMT expression has been previously observed (Pieper et al, 1991; Harris et al, 1994; von Wronski and Brent, 1994), whereas methylation at the 5′ promotor region has been associated with complete suppression of the gene (von Wronski et al, 1992; von Wronski and Brent, 1994). Consistent with the latter correlation, cells expressing high levels of MGMT, such as Daoy and U-138, are not methylated at the *Sma*I site of the promoter. It is not surprising that the *Sma*I site remained methylation free under conditions of MET deprivation, although such methylation would be consistent with the observed MGMT suppression. It is still possible that methylation of the promoter region at other sites could be involved in transcriptional suppression as a number of sites with such potential have been identified in tumour cells (Qian et al, 1995). However, regulation of MGMT activity by hypermethylation of the promoter is not expected to occur in a MET-deficient state, especially in the presence of high levels of HCY which could result in the accumulation of S-adenosylhomocysteine and consequently in the inhibition of DNA methylation and other transmethylation reactions (Johnson and Aswad, 1993). If MET deprivation regulates MGMT activity by changing the methylation status of the gene, it would be expected that such regulation would be the result of the hypomethylation of the downstream region rather than the methylation of the promoter. The absence of changes in the methylation of the body of the gene indicates that either methylation is not involved in the down-regulation of MGMT by MET deprivation, or changes of the status of methylation are not detected by the methodology used. If methylation of CpG islands is not involved in the down-regulation of MGMT activity in MET-dependent tumours by MET deprivation, it may be because of changes in chromatin structure or trans-acting factors by mechanisms not yet identified.

The down-regulation of MGMT by MET deprivation in *mer*$^+$, MET-dependent tumour cells indicates that MET-dependent tumours are susceptible to MET deprivation not only because of cell cycle blocks, but also because they are sensitized to alkylating agents. Our results indicate that the reduction of MGMT activity is not strictly dependent on the degree of sensitivity of the tumour to MET deprivation. Tumour cells that are moderately MET dependent and resist MET withdrawal, such as U-138, lose their MGMT activity nearly as fast as fully MET-dependent lines, such as the H-1623 and Daoy. This is particularly important in vivo where MET levels cannot be indefinitely suppressed to near zero levels. Thus, MET-depleting regimens, dietary or enzymatic, could be used to arrest or reverse tumour growth long enough to suppress MGMT activity. Elimination of a substantial portion of the tumour mass as a result of $G_2$ blocks may facilitate further treatment of the tumour, but it is not an absolute requirement for the employment of chemotherapy aiming to kill cells sensitized to DNA damage. Additional mechanisms of resistance may also have been compromised by the lack of MET, which could explain the reported susceptibility of MET-dependent cells to the combination of MET depletion with antineoplastic drugs other than alkylating agents. These results further support the use of MET depletion regimens in combination with genotoxic drugs for the treatment of MET-dependent, *mer*$^+$ tumours.

### ACKNOWLEDGEMENT

This study was supported in part by NIH grants NS20581 (SCS), CA23099 (TPB) and CA14799 (TPB).

### ABBREVIATIONS

MGMT, *O*$^6$ methylguanine-DNA methyltransferase; *O*$^6$-MeGua, *O*$^6$-methylguanine; 7-MeGua, 7-methylguanine; MET, methionine; HCY, homocysteine; DTT, 1, 4-dithiothreitol; BSA bovine serum albumin; *mer*$^{+/-}$, MGMT expressing/deficient; GAPDH, glyceraldehyde-3-phosphate dehydrogerase

### REFERENCES

Bradford M (1976) A rapid and sensitive method for the quantitation of microgram quantities of protein utilizing the principle of protein-dye binding. *Anal Biochem* **72**: 248–254

Branch P, Aquilina G, Bignami M and Karran P (1993) Defective mismatch binding and a mutator phenotype in cells tolerant to DNA damage. *Nature* **362**: 652–654

Breillout F, Hadida F, Enchinard-Garin P, Lascaux V and Poupon MF (1987) Decreased rat rhabdomyosarcoma pulmonary metastasis in response to a low methionine diet. *Anticancer Res* **7**: 861–867

Breillout F, Antoine E and Poupon MF (1990) Methionine dependency of malignant tumours: a possible approach for therapy. *J Natl Cancer Inst* **82**: 1628–1632

Brent TP, Von Wronski MA, Remack JS and Bigner DD (1991) *In vivo* stability of human O$^6$-methylguanine-DNA methyltransferase. *Am Assoc Cancer Res* **32**: 2505

Brent TP, Von Wronski MA, Edwards CC, Bromley M, Margison GP, Rafferty JA, Pegram CN and Bigner DD (1993) Identification of nitrosourea-resistant human rhabdomyosarcomas by in situ immunostaining of O$^6$-methylguanine-DNA methyltransferase. *Oncol Res* **5**: 83–86

Costello JF, Futsher BW, Kroes RA and Pieper RO (1994) Methylation-related chromatin structure is associated with exclusion of transcription factors from and suppressed expression of the O-6-methylguanine DNA methyltransferase gene in human glioma cell lines. *Mol Cell Biol* **14**: 6515–6521

Day RS III, Ziolkowski CH, Scudiero DA, Meyer SA, Lubiniecki AS, Girardi AJ, Galloway SM and Bynum GD (1980) Defective repair of alkylated DNA by human tumor and SV40 transformed human cell strains. *Nature* **288**: 724–727

Dolan ME, Stine L, Mitchell RB, Moschel RC and Pegg AE (1990) Modulation of mammalian O$^6$-alkylguanine-DNA alkyltransferase *in vivo* by O$^6$-benzylguanine and its effects of the sensitivity of a human glioma tumor to 1-(2-chloroethyl)-3-(4-methylcyclohexyl)-1-nitrosourea. *Cancer Commun* **2**: 371–377

Dolan ME, Pegg AE, Moschel RC and Grindley GB (1993) Effect of O$^6$-benzylguanine on the sensitivity of human colon tumor xenografts to 1, 3-bis (2-chloroethyl)-1-nitrosourea (BCNU). *Biochem Pharm* **46**: 285–290

Dunn WC, Foote RS, Hand RE and Mitra S (1986) Cell cycle dependent modulation of O$^6$-methylguanine-DNA-methyltransferase in C3H/10T1/2 cells. *Carcinogenesis* **7**: 807–812

Dunn WC, Tano K, Horesovsky J, Preston RJ and Mitra S (1991) The role of O$^6$-alkylguanine in cell killing and mutagenesis in Chinese hamster ovary cells. *Carcinogenesis* **12**: 83–89

Egyhazi S, Bergh J, Hansson J, Karran P and Ringborg U (1991) Carmustine-induced toxicity, DNA crosslinking and O$^6$-methylguanine-DNA methyltransferase activity in two human lung cancer cell lines. *Eur J Cancer* **27**: 1658–1662

Finkelstein JD (1990) Methionine metabolism in mammals. *J Nutr Biochem* **1**: 228–237

Fiskerstrand T, Christensen B, Tysnes OB, Ueland PM and Refsum H (1994) Development and reversion of methionine dependence in a human glioma cell line: relation to homocysteine remethylation and cobalamin status. *Cancer Res* **54**: 4899–4906

Gerson SL, Miller K and Berger NA (1985) O$^6$-alkylguanine-DNA alkyltransferase activity in human myeloid cells. *J Clin Invest* **76**: 2106–2114

Goseki N, Yamazaki S, Endo M, Onodera T, Kosaki G, Hibino Y and Kuwahata T (1992) Antitumor effect of methionine-depleting total parenteral nutrition with doxorubicin administration on Yoshida sarcoma-bearing rats. *Cancer* **69**: 1865–1872

Guo H, Herrera H, Groce A and Hoffman RM (1993*a*). Expression of the biochemical defect of methionine dependence in fresh patient tumors in primary histoculture. *Cancer Res* **53**: 2479–2483

Guo H, Lishko VK, Herrera H, Groce A, Kubota T and Hoffman RM (1993*b*). Therapeutic tumor-specific cell cycle block induced by methionine starvation *in vivo*. *Cancer Res* **53**: 5676–5679

© Cancer Research Campaign 1997

Halpern BC, Clark BR, Hardy DN Halpern RM and Smith RA (1974) The effect of replacement of methionine by homocysteine on survival of malignant and normal adult mammalian cells in culture. *Proc Natl Acad Sci USA* **71**: 1133–1136

Harris LC, Potter PM, Tano K, Shiota S Mitra S and Brent TP (1991) Characterization of the promoter region of the human O$^6$-methylguanine-DNA methyltransferase gene. *Nucleic Acids Res* **19**: 6163–6167

Harris LC, Remack JS and Brent TP (1994) In vitro methylation of the human O$^6$-methylguanine-DNA methyltransferase promoter reduces transcription. *Biochim Biophys Acta* **1217**: 141–146

Hoffman RM (1990) Unbalanced transmethylation and the perturbation of the differentiated state leading to cancer. *BioEssays* **12**: 163–166

Hoffman RM and Erbe RW (1976) High in vivo rates of methionine biosynthesis in transformed human and malignant rat cells auxotrophic to methionine. *Proc Natl Acad Sci USA* **73**: 1523–1527

Hori H, Takabayashi K, Orvis L, Carson DA and Nobori T (1996) Gene cloning and characterization of *Pseudomonas putida* L-methionine-alpha-deamino-gamma-mercaptomethane-lyase. *Cancer Res* **56**: 2116–2122

Hoshiya Y, Guo H, Kubota T, Inada T, Asanuma F, Yamada Y, Koh J, Kitajima M and Hoffman RM (1995) Human tumors are methionine dependent *in vivo*. *Anticancer Res* **15**: 717–718

Jackson RC (1992) Cell cycle effects of drugs. In *The Theoretical Foundations of Cancer Chemotherapy Introduced by Computer Models* RC Jackson (ed.), pp. 1–45. Academic Press: San Diego

Johnson BA and Aswad DW (1993) Kinetic properties of bovine brain protein L-isoaspartyl methyltransferase determined using a synthetic isoaspartyl peptide substrate. *Neurochem Res* **18**: 87–94

Judde JG, Ellis M and Frost P (1989) Biochemical analysis of the role of transmethylation in the methionine dependence state. *Cancer Res* **49**: 4859–4865

Kokkinakis DM, Moschel RC, Vuong TH, Reddy MV, Schold SC and Pegg AE (1996) Mechanism of depletion of O$^6$-Methylguanine-DNA methyltransferase activity in rat tissues by O$^6$-benzyl-2′-deoxyguanosine. Role of metabolism. *Anticancer Res* **16**: 1–10

Kroes RA and Erickson LC (1995) The role of mRNA stability and transcription on 06-methylguanine-DNA methyltransferase (MGMT) expression in Mer– human tumor cells. *Carcinogenesis* **16**: 2255–2257

Kubota R, Kubota K, Yamada S, Tada M, Takahashi T, Iwata R and Tamahashi N (1995) Methionine uptake by tumor tissue: a microautoradiographic comparison with FDG. *J Nucl Med* **36**: 484–492

Laemmli UK (1970) Cleavage of structural proteins during assembly of the head of bacteriophage T4. *Nature* **27**: 680–685

Lishko VK, Lishko OV and Hoffman RM (1993) Depletion of serum methionine by methioninase in mice. *Anticancer Res* **13**: 1465–1468

Liteplo RG (1990) Reversion to a homocysteine-responsive phenotype in a human melanoma cell line is associated with diminished growth potential and increased methionine biosynthesis. *Exp Cell Res* **186**: 340–345

Liteplo RG, Hipwell SE, Rosenblatt DS, Sillaots S and Lue-Shing H (1991) Changes in cobalamin metabolism are associated with the altered methionine auxotrophy of highly growth autonomous human melanoma cells. *J Cell Physiol* **149**: 332–338

Marathi UK, Kroes RA, Dolan ME and Erickson LC (1993) Prolonged depletion of O$^6$-methylguanine DNA methyltransferase activity following exposure to O$^6$-benzylguanine with or without streptozotocin enhances 1,3-bis(2-chloroethyl)-1-nitrosourea sensitivity *in vitro*. *Cancer Res* **53**: 4281–4286

Matsudaira P (1987) Sequence from picomole quantities of proteins electroblotted onto polyvinylidene difluoride membranes. *J Biol Chem* **262**: 10035–10038

Mineura K, Sasajima T, Kuwahara N, Kowada M, Murakami M and Uemura K (1993) (14C-methyl)-L-methionine uptake in rat brain tumors before and after treatment with the protein synthesis inhibitor cycloheximide. *J Neuro-Oncol* **15**: 229–233

Pegg AE (1984) S-adenosylmethionine decarboxylase: a brief review. *Cell Biochem Funct* **2**: 11–15

Pieper RO, Costello JF, Kroes RA, Futscher BW, Marathi UK and Erickson LC (1991) Direct correlation between methylation status and expression of the human O$^6$-methylguanine DNA methyltransferase gene. *Cancer Comm* **3**: 241–253

Qian X, Von Wronski MA and Brent TP (1995) Localization of methylation sites in the human O$^6$-methylguanine-DNA methyltransferase promoter: correlation with gene suppression. *Carcinogenesis* **16**: 1385–90

Sambrook J, Fritsch EF and Maniatis T (1989) *Molecular Cloning: A Laboratory Manual* 2nd edn. Cold Spring Harbor Laboratory Press:Cold Spring Harbor

Schold JR SC, Friedman HS, Brent TP, Bigner SH and Bigner DD (1989) O$^6$-alkylguanine-DNA alkyltransferase and sensitivity to procarbazine in human tumor xenografts. *J Neurosurg* **70**: 573–577

Schold SC JR, Kokkinakis DM, Rudy JL, Moschel RC and Pegg AE (1996) Treatment of human brain tumor xenografts with O$^6$-benzyl-2′-deoxyguanosine and BCNU. *Cancer Res* **56**: 2076–2081

Stern PH and Hoffman RM (1984) Elevated rates of transmethylation in cell lines from diverse human tumors. *In Vitro Rapid Commun Cell Biol* **20**: 663–670

Stern PH and Hoffman RM (1986) Enhanced in vitro selective toxicity of chemotherapeutic agents for human cancer cells based on a metabolic defect. *J Natl Cancer Inst* **76**: 629–639

Tano K, Shiota S, Collier J, Foote RS and Mitra S (1990) Isolation and structural characterization of a cDNA clone encoding the human repair protein for O$^6$-alkylguanine. *Proc Natl Acad Sci USA* **87**: 686–690

Tautt JW, Anuszewska EL and Koziorowska JH (1982) Methionine regulation of *N*-5-methyltetrahydrofolate: homocysteine methyltransferase and its influence on the growth and protein synthesis in normal, neoplastic, and transformed cells in culture. *J Natl Cancer Inst* **69**: 9–14

Tsujimura T, Zhang Y, Fujio C, Chang HR, Watanani M, Ishizaki K, Kitamura H and Ikenaga M (1987) O$^6$-methylguanine methyltransferase activity and sensitivity of Japanese tumor cell strains to 1-(4-amino-2-methyl-5-pyrimidinyl)methyl-3-(2-chloroethyl)-3-nitrosourea hydrochloride. *Jap Cancer Res* **78**: 1207–1215

Vanhamme L and Szpirer C (1989) Spontaneous and 5-azacytidine-induced revertants of methionine-dependent tumor-derived and H-*ras*-1-transformed cells. *Exp Cell Res* **181**: 159–168

Von Wronski M and Brent T (1994) Effect of 5-azacytidine on expression of the human DNA repair enzyme O$^6$-methylguanine-DNA methyltransferase. *Carcinogenesis* **15**: 577–582

Von Wronski MA, Harris LC, Tano K, Mitra S, Bigner DD and Brent TP (1992) Cytosine methylation and suppression of O$^6$-methylguanine-DNA methyltransferase expression in human rhabdomyosarcoma cell lines and xenografts. *Oncol Res* **4**: 167–174

Wang Y, Kato T, Ayaki H, Ishizaki K, Tano K, Mitra S and Ikenaga M (1992) Correlation between DNA methylation and expression of O$^6$-methylguanine-DNA methyltransferase gene in cultured human tumor cells. *Mutation Res* **273**: 221–230

© Cancer Research Campaign 1997